# Discovery of a Novel, Monocationic, Small-Molecule Inhibitor of Scrapie Prion Accumulation in Cultured Sheep Microglia and Rov Cells

**DOI:** 10.1371/journal.pone.0051173

**Published:** 2012-11-30

**Authors:** James B. Stanton, David A. Schneider, Kelcey D. Dinkel, Bethany F. Balmer, Timothy V. Baszler, Bruce A. Mathison, David W. Boykin, Arvind Kumar

**Affiliations:** 1 Department of Veterinary Microbiology and Pathology, Washington State University, Pullman, Washington, United States of America; 2 United States Department of Agriculture, Agricultural Research Service, Animal Disease Research Unit, Pullman, Washington, United States of America; 3 Department of Chemistry, Georgia State University, Atlanta, Georgia, United States of America; Dulbecco Telethon Institute and Mario Negri Institute for Pharmacological Research, Italy

## Abstract

Prion diseases, including sheep scrapie, are neurodegenerative diseases with the fundamental pathogenesis involving conversion of normal cellular prion protein (PrP^C^) to disease-associated prion protein (PrP^Sc^). Chemical inhibition of prion accumulation is widely investigated, often using rodent-adapted prion cell culture models. Using a PrP^Sc^-specific ELISA we discovered a monocationic phenyl-furan-benzimidazole (DB772), which has previously demonstrated anti-pestiviral activity and represents a chemical category previously untested for anti-prion activity, that inhibited PrP^Sc^ accumulation and prion infectivity in primary sheep microglial cell cultures (*PRNP* 136VV/154RR/171QQ) and Rov9 cultures (VRQ-ovinized RK13 cells). We investigated potential mechanisms of this anti-prion activity by evaluating PrP^C^ expression with quantitative RT-PCR and PrP ELISA, comparing the concentration-dependent anti-prion and anti-pestiviral effects of DB772, and determining the selectivity index. Results demonstrate at least an approximate two-log inhibition of PrP^Sc^ accumulation in the two cell systems and confirmed that the inhibition of PrP^Sc^ accumulation correlates with inhibition of prion infectivity. *PRNP* transcripts and total PrP protein concentrations within cell lysates were not decreased; thus, decreased PrP^C^ expression is not the mechanism of PrP^Sc^ inhibition. PrP^Sc^ accumulation was multiple logs more resistant than pestivirus to DB772, suggesting that the anti-PrP^Sc^ activity was independent of anti-pestivirus activity. The anti-PrP^Sc^ selectivity index in cell culture was approximately 4.6 in microglia and 5.5 in Rov9 cells. The results describe a new chemical category that inhibits ovine PrP^Sc^ accumulation in primary sheep microglia and Rov9 cells, and can be used for future studies into the treatment and mechanism of prion diseases.

## Introduction

Prion diseases (transmissible spongiform encephalopathies [TSEs]) are progressive, fatal, transmissible, neurodegenerative diseases, which include scrapie in sheep and goats, bovine spongiform encephalopathy (BSE) in cattle, chronic wasting disease (CWD) in deer and elk, and various forms of Creutzfeldt-Jakob disease (CJD) and kuru in humans [1]. The similarities between scrapie and CJD have long been recognized [2], and scrapie is the prototypical prion disease [3]; thus, scrapie is an experimental model that allows for the investigation of a natural prion disease in a natural host. The central feature of prion pathogenesis is the conversion of the normal cellular form of the host-encoded prion protein (PrP^C^ [C superscript for cellular]) to an abnormal isoform, designated PrP^Sc^ (Sc superscript for sheep scrapie) [4], [5], [6]. This post-translational conversion involves a conformational change resulting in a detergent-insoluble, partially protease-resistant molecule that aggregates in affected cells and serves as the marker for prion diseases. PrP^Sc^-accumulating cells include neurons and monocyte-derived cells (macrophages, microglia, and dendritic cells), among others [7], [8], [9], [10], [11].

Studies to identify anti-prion compounds often initially rely on inhibition of in vitro PrP^Sc^ formation [12]. Previous categories of compounds that have demonstrated anti-PrP^Sc^ activity in cell lines or animals include sulfated polyanions (e.g., pentosan polysulfate, dextran sulfate) [13], [14], [15], [16], [17], [18], sulfonated dyes (e.g., congo red) [19], [20], [21], [22], cyclic tetrapyrroles (e.g., porphyrins) [23], [24], [25], [26], polyene antibiotics (e.g., amphotericin B) [27], [28], [29], [30], [31], branched polyamines [32], [33], quinolones and tricyclics (e.g., quinacrine) [12], [34], [35], [36], [37], [38], polyphenols (e.g., tannins) [12], statins (e.g., lovastatin) [12], 2-aminothiazoles [39], and phosphorothioate oligonucleotides [40], [41], [42]. Currently, however, there are no effective treatments for prion diseases despite abundant investigation into therapeutics [43], [44], [45]. Continued investigation into new classes of anti-prion compounds is thus warranted, not only for the development of effective in vivo anti-prion molecules, but also as research tools to elucidate the cellular pathogenesis of prion diseases.

Most of the studies to detect anti-prion compounds have used rodent cell culture systems with rodent-adapted prion strains. While these rodent models have many benefits, attempts have been made at improving upon them. Rov9 cells are rabbit renal epithelial cells (RK-13) that have the 136VV/154RR/171QQ allele of the sheep PRNP gene under control of a doxycycline-inducible promoter and accumulate sheep-derived prions [46]. Using these more natural, yet still far from completely natural, cells it has been shown that anti-prion compounds identified using rodent-adapted PrP^Sc^ systems often fail to demonstrate anti-prion activity when using sheep-origin PrP^Sc^ [47]. The inability of these compounds to specifically inhibit sheep-derived prions suggests the importance for even more natural prion models for anti-prion compound screening as the species of origin or cell type may also impact the results. Currently there are only two cell culture models that are derived from a natural TSE host, a mule deer-derived brain fibroblast cell line susceptible to PrP^CWD^ [48] and a sheep-derived microglial cell system susceptible to sheep-origin PrP^Sc^ [49]. The mule deer-derived brain fibroblast cell line has been used to demonstrate the anti-prion activity of pentosan polysulfate and a porphyrin compound [48].

Besides the varying effectiveness of anti-prion compounds in different systems, another consideration for model development is the potential for significant effects of co-infecting agents. It has been shown that small ruminant lentivirus infection is associated with enhanced distribution of PrP^Sc^ in naturally co-infected sheep [50], [51]. This effect in sheep may be related to virus-enhanced, intracellular accumulation of PrP^Sc^, as has been demonstrated in vitro using primary sheep microglial cells [49]. It is unknown if other virus families have similar effects.

**Figure 1:**
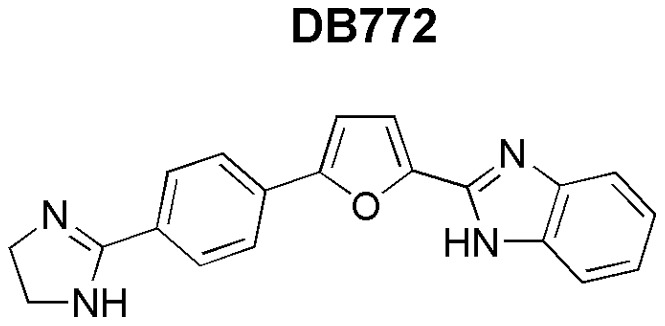
Structure of 2-(2-benzimidazolyl)-5-[4-(2-imidazolino)phenyl]furan dihydrochloride (DB772).

Flaviviruses are a group of enveloped, positive-sense stranded RNA viruses that can infect monocyte-lineage cells, establish persistent infections in vivo, and establish noncytopathic infections in vitro [52], [53], [54]. Rov9 cells, as derivatives of RK13 cells [55], and sheep microglial cells (data reported herein) are susceptible to bovine viral diarrhea virus (BVDV, genus *Pestivirus*, family *Flaviviridae* ) infection. To cure cells of a potentially confounding, co-infecting virus, 2-(2-benzimidazolyl)-5-[4-(2-imidazolino)phenyl]furan dihydrochloride (DB772; Fig. 1), a known BVDV inhibitor [56], [57], was used. In addition to inhibiting BVDV, this treatment also inhibited PrP^Sc^ accumulation. Here we describe the anti-prion activity of DB772, a monocationic phenyl-furan-benzimidazole [58], which belongs to a chemical category previously untested for anti-PrP^Sc^ activity.

In summary, only one study has investigated in vitro chemical inhibition of prions in a cell system derived from a natural host [48] and no studies have tested for anti-prion activity in a sheep cell culture system or in microglial cells from any species, despite the relevance of sheep scrapie and monocyte-derived cells (e.g., microglia) to prion diseases. Reported herein is the discovery of anti-prion activity of a compound belonging to a previously untested chemical category using sheep-origin PrP^Sc^ and sheep microglial cells.

## Materials and Methods

### Ethics Statement

The Institutional Animal Care and Use Committee at Washington State University approved this study protocol (Permit numbers: #03811 and 03987). The ewe was euthanized by administering an intravenous overdose of sodium pentobarbital, in accordance with the 2007 American Veterinary Medical Association Guidelines on Euthanasia, and all efforts were made to minimize suffering.

### Cells

Primary sheep microglial cells were obtained from a near-term Suffolk-cross fetus and cultured as previously described [49]. All cell media were made with pestivirus-free, fetal-bovine serum. Microglial cells were phenotyped via immunocytochemistry using the microglial markers biotinylated *Ricinus communis* agglutinin-1 (RCA-1) (Dako Cytomation) and an anti-CD14 antibody (MM61A, IgG1, VMRD, Inc.), as previously described [49]. A pellet of microglial cells was collected, washed by centrifugation, and used for genotyping the fetal prion gene as previously described [59].

Rov9 cells (B. Caughey with permission from D. Vilette) are rabbit renal epithelial cells (RK-13) stably transfected with the sheep VRQ (Val-136, Arg-154, Gln-171) allele of the prion gene under the control of a tetracycline-inducible promoter [46]. Rov9 cells were maintained in OMEM supplemented with 1 µg/ml doxycycline (OMEM-Doxy), as previously described [46]. PrP^Sc^ within Rov9^Sc^ cells was verified by PrP^Sc^-specific enzyme-linked immunosorbent assay (ELISA) (see below).

Since Rov9 cells are derived from RK13 cells, Rov9 cells are permissive to BVDV infection [55]. Prior to inoculation microglial cells were confirmed BVDV negative and Rov9 cells were confirmed BVDV positive by RT-PCR and BVDV antigen ELISA (see below). The scrapie inoculum also contains infectious BVDV, and the preparation and application of PrP^Sc^ inoculum also transmits BVDV. Untreated microglial cells were used as controls for BVDV contamination.

### Inoculation with PrP^Sc^

For use as an inoculum, mechanical lysates of the Rov9^Sc^ (Rov9 cells infected with PrP^Sc^) and Rov9^C^ (Rov9 cells not infected with PrP^Sc^) cells were prepared as previously described [46]. Briefly, the Rov9 cells were grown to confluence in two 75-cm^2^ plastic tissue culture flasks. Rov9^Sc^ and Rov9^C^ cells were rinsed three times with sterile 1× Dulbecco's-PBS (D-PBS) and scraped into 10 ml of PBS. The cell pellets were collected by centrifugation at 220×*g* at room temperature for 7 min and resuspended in 0.5 ml of filter-sterilized 5% glucose. The cell suspensions were frozen and thawed four times and then subjected to 1 to 2 min of sonication in a cup horn sonicator. The inoculum was stored at –20°C. For inoculation, cells were passed into twelve-well plates and allowed to grow to approximately 60% confluence. Cells were rinsed once with PBS and then overlaid with 200 µl of a 1/20 dilution of either the Rov9^Sc^ lysate (microglia^Sc^) or the Rov9^C^ lysate (microglia^C^) in OPTI-MEM. Microglia^Sc^ (or Rov9^Sc^) and microglia^C^ (or Rov9^C^) were incubated for 6 hours, and then 200 µl of OMEM (or OMEM-Doxy) was added to each well. Following an additional 2 days of incubation, 0.5 ml of appropriate medium was added to each well, and cells were incubated for 4 days at which time they were expanded into 25-cm^2^ tissue culture flasks. Cells were fed every 3 or 4 days with appropriate medium as necessary and serially passaged 1/5 after reaching confluence.

### DB772 inhibits PrP^Sc^ accumulation and prion infectivity

To eliminate BVDV from cultures 2-(2-benzimidazolyl)-5-[4-(2-imidazolino)phenyl]furan dihydrochloride (DB772) was used at 4 μM, as this is the concentration that cured primary bovine fibroblasts of pestivirus infection [56]. A 10 mM stock solution of DB772 was prepared in sterile water and stored at –20°C until use. Rov9^Sc^, Rov9^C^, microglia^Sc^, and microglia^C^ were each split and cultured separately. There were 8 final treatment groups (Rov9^Sc/DB772^, Rov9^Sc/UnTx^, Rov9^C/DB772^, Rov9^C/UnTx^, microglia^Sc/DB772^, microglia^ Sc/UnTx^, microglia^C/DB772^, and microglia^C/UnTx^ [UnTx stands for untreated]). DB772 was maintained in the appropriate culture medium for four passages. Cells were then grown for an additional four passages without DB772 in the culture medium. Cells were collected after four passages with DB772 (P-4, “end of DB772 Tx”) and after four additional passages without DB772 (P-8, “end of clearance”) for pestivirus quantification, PrP^Sc^ quantification, and PrP^C^ quantification (see below). Mechanical lysates of Rov9^Sc/DB772^ were also collected for prion inoculum creation (see above) to verify that prion infectivity was also inhibited by DB772.

### DB772 concentration-dependence curve

To compare the concentration dependencies of BVDV and PrP^Sc^, microglia^Sc^ and Rov9^Sc^ cells were treated with a dilution series of DB772. Samples were collected and analyzed for BVDV ELISA and PrP^Sc^ ELISA as described below. Curcumin (Sigma-Aldrich, St. Louis, MO, USA), which does not inhibit PrP^Sc^ accumulation in Rov9^Sc^ cells [47], was used as a negative control for non-specific cell death-induced PrP^Sc^ inhibition. Three independent experiments were analyzed. The 50% tissue culture effective concentration (TCEC_50_) was determined by nonlinear regression using a four-parameter logistic model for microglia^Sc^ and a two-parameter exponential decay model for Rov^Sc^ (SigmaPlot ver. 11).

### Sample collection

Cells in two 75-cm^2^ flasks for each treatment group were trypsinized, resuspended, counted by light microscopy using a cytometer, and aliquoted appropriately for each assay.

For ELISAs (BVDV, PrP^Sc^, and total PrP), total protein evaluation, and RNA extraction, cells were collected by centrifugation at 220×*g* at room temperature for 7 min. The supernatant was aspirated and the cell pellet washed in D-PBS. Cells were collected again by centrifugation at 220×*g* at room temperature for 7 min, and the supernatant was aspirated.

For ELISAs and total protein measurements, cells were lysed in lysis buffer (0.5% Triton X-100, 0.5% sodium deoxycholate, 50 mM Tris-HCl [pH 8.0], 5 mM EDTA, and 150 mM NaCl) for 3 min at room temperature with gentle rocking, followed by centrifugation at 2,300×*g* at room temperature for 5 min. Cell lysates were stored at –20°C until evaluation.

For RT-PCR, the washed cell pellet was lysed in buffer QLT (Qiagen, Valencia, CA, USA). Cell lysates were shredded (QiaShredder, Qiagen) and total RNA was purified using RNeasy mini spin columns (Qiagen) following manufacturer's directions. Total RNA quantity and quality was determined by spectrophotometry (Thermo Scientific, ND-1000).

### Total protein measurement

Since cells were collected based on a fraction of the cell suspension, total protein was analyzed to normalize samples for the various ELISAs. Aliquots of the cell lysates were diluted 1/5 into lysis buffer and then assayed for total protein using the bicinchoninic acid (BCA) protein assay reagent (Thermo Fisher Scientific Inc, Rockford, IL, USA) following manufacturer's directions for the microplate-based assay.

### Pestivirus measurement

Pestivirus infection in cells was confirmed by RT-PCR and enzyme-linked immunosorbent assay (ELISA). To detect pestiviral RNA, 50 ng of total RNA was reverse transcribed into cDNA using random hexamers (SuperScript III First-Strand Synthesis System, Invitrogen, Carlsbad, CA, USA). The resulting cDNA was amplified using previous methods [60]. Briefly, DNA was amplified in a reaction consisting of 1 μl of cDNA template, 2 μM of each primer (Table 1), 12.5 μl of JumpStart^TM^ REDTaq® ReadyMix^TM^ Reaction Mix (Sigma-Aldrich, St. Louis, MO, USA) and nuclease-free water to a final volume of 25 μl. The reaction was incubated in a thermocycler (C1000, Bio-Rad, Hercules, CA, USA) under the following conditions: 95°C/5 min; 35 cycles of 95°C/30 sec, 56°C/30sec, 72°C/30 sec; and 72°C/5 min. Isolation of detectable amounts of RNA was confirmed by amplifying glyceraldehyde 3-phosphate dehydrogenase (GAPDH) mRNA using intron-spanning primers (Table 1) [61] under the same PCR conditions described above. Amplification products were separated by agarose gel electrophoresis and visualized by staining with ethidium bromide. BVDV-free bovine turbinate cells were cultured in parallel with the Rov9 and microglial cells and served as negative controls.

**Table 1:** RT-PCR primer information.

Target	Forward primer sequence	Reverse Primer Sequence	Amplicon size (bp)	Reference
BVDV^a^	ATGCCCT/ATAGTAGGACTAGCA	TCAACTCCATGTGCCATGTAC	288	[60]
GAPDH^a^	GAGATGATGACCCTTTTGGC	GTGAAGGTCGGAGTCAACG	353	[61]
PrPC^b^	CCGTTACCCCAACCAAGTGT	CCGTTACCCCAACCAAGTGT	159	NA
GAPDH-Ov^b^	GGCGTGAACCACGAGAAGTATAA	CCCTCCACGATGCCAAAGT	120	[69]
GAPDH-Rab^b^	GCCATCACTGCCACCCAG	GAGTTTCCCGTTCAGCT	147	NA

a RT-PCR primer set. ^b^qRT-PCR primer set.

BVDV antigen was detected by commercial ELISA (HerdChek^*^, BVDV Antigen Test Kit, IDEXX, Westbrook, ME, USA) following the manufacturer's directions. Aliquots of cell lysates were diluted appropriately in lysis buffer to normalize protein concentrations (based on BCA results) and then added to the kit ELISA plate. The proprietary ELISA positive and negative controls were used per the manufacturer's directions. Corrected optical densities were transformed to sample/positive ratio (S/P) using manufacturer's directions: (sample-negative)/(positive-negative). A standard curve prepared from a half-log dilution series of BVDV-infected cell lysate was used to transform the S/P results to relative concentration of BVDV antigen. Untreated, prion-inoculated lysates at passage 4 were set at log 1, and all other results were normalized to this value. The relative amount of BVDV antigen within each passage was compared based on DB772 treatment status and PrP^Sc^ inoculation status using a two-way ANOVA followed by the Holm-Sidak method of multiple comparisons with a cutoff *P* value of 0.05 (SigmaPlot 11.2.0.5).

### PrP^Sc^ ELISA and immunoblot assays

PrP^Sc^ was detected by commercial ELISA (HerdChek^*^, Scrapie Antigen Test Kit, IDEXX) following the manufacturer's directions. Aliquots of cell lysates were diluted appropriately in lysis buffer to normalize protein concentrations (based on BCA results) and then added to the kit ELISA plate. The proprietary ELISA positive and negative controls were used per the manufacturer's directions. A standard curve prepared from a half-log dilution series of Rov9^Sc^ cell lysate was used to transform corrected optical density results to relative PrP^Sc^ concentrations. Untreated, prion-inoculated lysates at passage 4 were set at log 1, and all other results were normalized to this value. PrP^Sc^ concentrations within each passage were compared based on DB772 treatment status using an independent *t* test with a cutoff *P* value of 0.05 (SigmaPlot).

The specificity for samples derived from cultured sheep microglial cells and Rov9 cells has been previously confirmed by comparison with proteinase K-resistant immunoblotting [49]. In the present study, Rov9 cells were collected at passage five with and without 4 μM DB772 treatment (Rov9^Sc/UnTx^ and Rov9^Sc/DB772^) for proteinase K treatment (50 μg/ml [2 units/ml]), phosphotungstic acid (PTA) precipitation, and immunoblotting (primary antibody F99/97.6.1), as previously described, [49] to again confirm that loss of ELISA signal was associated with loss of a proteinase K-resistant prion-specific protein band. Replicate aliquots of PTA-precipitated samples were electrophoresed and stained, following manufacturer's directions, with SYPRO Ruby (Sigma-Aldrich) to confirm the precipitation of protein in the untreated and the DB772-treated samples.

### *PRNP* and PrP^C^ measurement

*PRNP* transcript levels were quantified using quantitative RT-PCR. RNA samples were treated with DNase (DNA-free kit, Ambion, Austin, TX, USA) followed by DNase Inactivation Reagent (Ambion) and centrifugation at 10,000×*g* for 1.5 min. Each treatment sample was tested in triplicate. One microgram of RNA was reverse transcribed into cDNA using the SuperScript III First-Strand Synthesis Supermix for qRT-PCR (Invitrogen). Quantitative real-time PCR was performed in an iCycler iQ (Bio-Rad). The 20-μl reaction contained 1× SYBR GreenER qPCR SuperMix for iCycler (Invitrogen), 200 nM of each specific primer (Table 1), 8 μl of 1/100 diluted cDNA (1 μg), and water. Reaction conditions were 50°C for 2 min, 95°C for 8.5 min, 40 cycles of denaturation at 95°C for 15 s and annealing at 59°C for 1 min followed immediately by a melt curve. A standard curve was used to transform C_T_ values to relative concentrations and results were expressed as log_2_ change (DB772-treated/Untreated). During validation for the newly designed RT-PCR primers, amplicons were sequenced by an ABI Prism 3730 DNA Analyzer with Big Dye Terminator chemistry (PE-Applied Biosystems).

Total PrP was detected by commercial ELISA (TeSeE^TM^ SAP Detection Kit, Bio-Rad) following the manufacturer's directions, except for omission of the proteinase K digestion step; thus, PrP^C^ was maintained within the samples. Aliquots of cell lysates were diluted appropriately (based on BCA results) so that a standard concentration of protein was added to the kit ELISA plate. The proprietary ELISA positive and negative controls were used per the manufacturer's directions. A standard curve prepared from a half-log dilution series of Rov^C^ cell lysate was used to transform corrected optical density results to relative PrP^C^ concentrations. Results were expressed as log_2_ change (DB772-treated/untreated).

For *PRNP* transcripts and total PrP levels, the log_2_ change was compared to the null hypothesis (no change) using individual one-sample *t* tests. A cutoff *P* value of 0.05 was used followed by the Bonferroni method for correcting for multiple comparisons (SigmaPlot).

### Cytotoxicity evaluation

Cytotoxicity was measured in exponentially growing cell cultures. Two thousand cells were plated into a flat-bottomed 96-well plate for 24 h prior to addition of half-log dilutions of DB772. Cell viability, expressed as a percentage of untreated control, was determined after four days using 2-(4-Iodophenyl)-3-(4-nitrophenyl)-5-(2,4-disulfophenyl)-2H-tetrazolium (WST-1) (Roche, United States), following manufacturer's directions. The 50% cytotoxic (CC_50_) for microglia and Rov9 cells were determined from four independent experiments by nonlinear regression using a four-parameter logistic model (SigmaPlot). The percent cell viability at 4 μM, at the TCEC_50_ (microglia: 2.4 μM; Rov9: 1.9 μM), and at 0 μM was compared within each passage, accounting for DB772-treatment status and PrP^Sc^-inoculation status, using a two-way ANOVA followed by the Holm-Sidak method of multiple comparisons with a cutoff *P* value of 0.05 (SigmaPlot).

## Results

### DB772 effect on pestivirus

**Figure 2:**
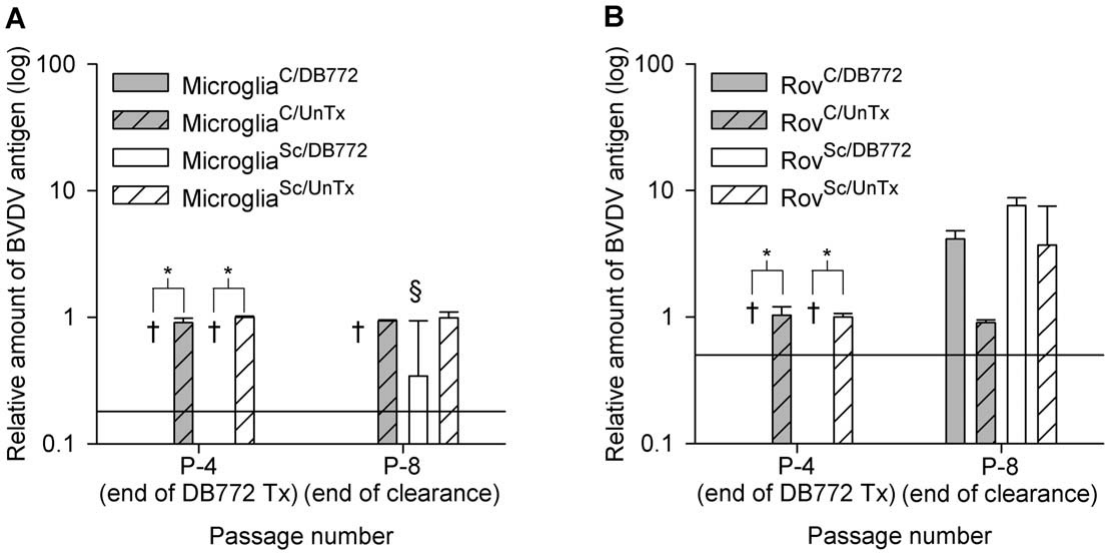
DB772 inhibits BVDV in primary sheep microglia (A) and Rov9 cells (B). Cells were inoculated with BVDV-containing PrP^Sc^ inoculum [55]. Following establishment of PrP^Sc^ accumulation, treatment groups were maintained in culture with 4 μM of DB772 for four passages. At the fourth passage (P-4, end of DB772 Tx), an aliquot of cells was lysed for BVDV antigen ELISA. All cultures were then continued for another four passages without DB772. Cells were collected for BVDV antigen ELISA at the end of those four clearance passages (P-8, end of clearance). A standard curve was used to transform the corrected optical densities into relative concentration of BVDV antigen. Data columns represent the means ± one standard deviation. Results at each passage were statistically compared between DB772-treated and untreated groups. The y-axis reference line indicates the minimum detection limit of the ELISA. *, *P*<0.001. †, below assay detection limit. §, one positive sample.

To create BVDV-free cells, sheep microglial cells and ovinized rabbit epithelial cells (Rov9), both of which accumulate sheep-origin PrP^Sc^, were cultured in the presence of 4 μM DB772 for four passages. Continuous DB772 treatment of microglial cells (Fig. 2A; P-4) and Rov9 cells (Fig. 2B; P-4) for four passages significantly (*P*<0.001) reduced BVDV antigen to below detectable limits. Based on the relative minimum detection limits of the BVDV ELISA, as determined by the standard curves, the decrease in BVDV antigen is at least 0.8 logs in sheep microglial cells and 0.3 logs in Rov9 cells. Due to the relative insensitivity of the BVDV ELISA and the lack of detectable BVDV antigen, the absolute fold change may be higher. The negative BVDV ELISA results were validated by BVDV-specific RT-PCR, which failed to amplify a product from any of the DB772-treated groups (data not shown). Curing of BVDV from all replicates was not accomplished as after four additional passages in the absence of DB772 (P-8), BVDV antigen (Fig. 2) and nucleic acid (data not shown) returned to detectable levels in one DB772-treated microglial sample (Fig. 2A; P-8) a single microglia^Sc/DB772^ sample) and in all of the Rov9^Sc/DB772^ and Rov9^C/DB772^ replicates (Fig. 2B; P-8). No influence of PrP^Sc^ status on anti-pestivirus effectiveness was detected.

### DB772 effect on PrP^Sc^

**Figure 3:**
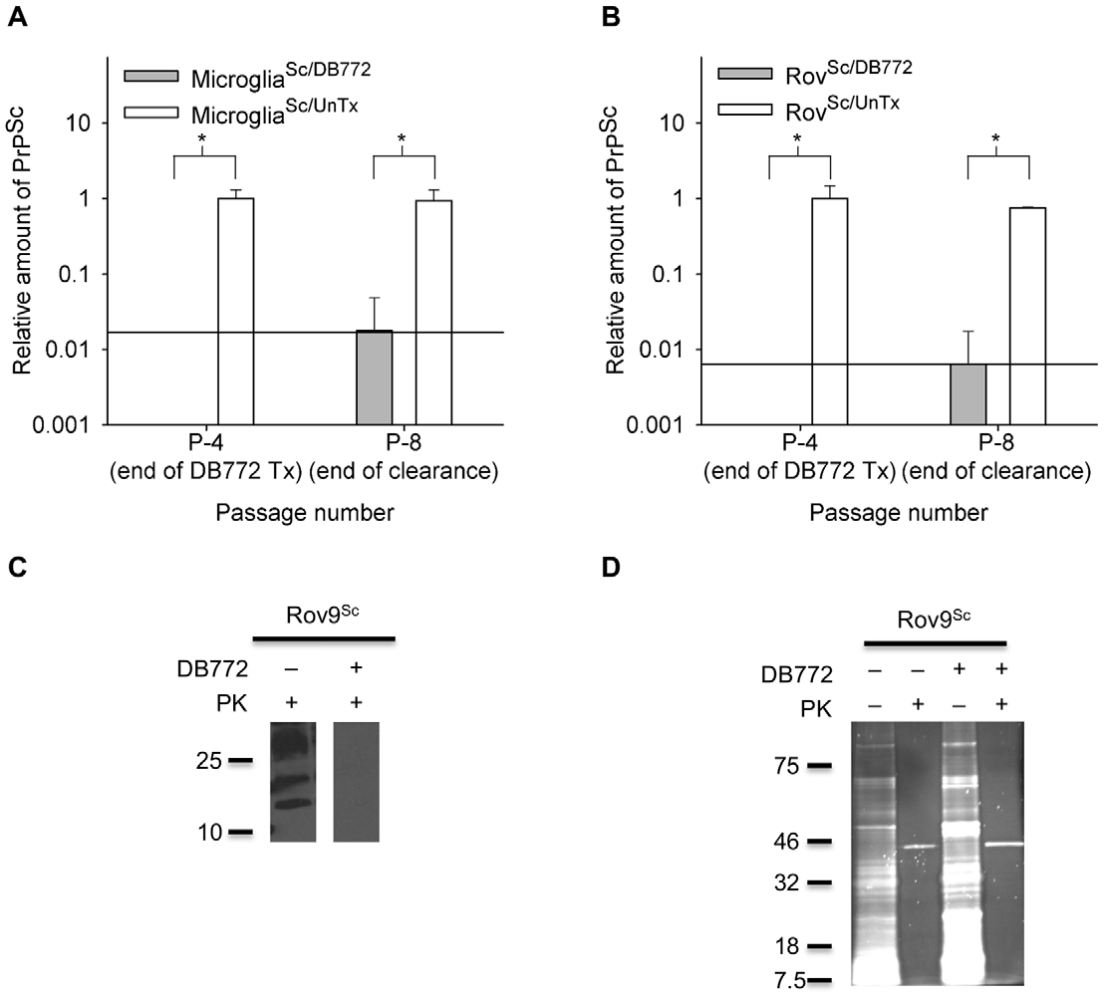
DB772 inhibits PrP^Sc^ accumulation in primary sheep microglial cells and Rov9 cells. Determination of PrP^Sc^ levels in sheep microglial cells (A) and Rov9 cells (B). Cells were inoculated with PrP^Sc^, and following establishment of PrP^Sc^ accumulation, treatment groups were maintained in culture with 4 μM of DB772 for four passages. At the fourth passage (P-4, end of DB772 Tx), an aliquot of cells was lysed for PrP^Sc^ ELISA. All cultures were then continued for another four passages without DB772. Cells were collected for PrP^Sc^ ELISA at the end of those four passages (P-8, end of clearance). A standard curve was used to transform the corrected optical densities into relative concentration of PrP^Sc^. Data columns represent the means ± one standard deviation. Results at each passage were statistically compared between DB772-treated and untreated groups. The y-axis reference line indicates the minimum detection limit of the ELISA. *, *P*<0.05. Positive and negative ELISA results were compared to proteinase K digestion-based immunoblotting (C). Passage 5 Rov9^Sc/UnTx^ and Rov9^Sc/DB772^ samples were lysed, treated with 50 μg/ml of proteinase K, precipitated with phosphotungstic acid, and immunoblotted using the monoclonal anti-PrP antibody F99/97.6.1. Proteinase K-resistant prion protein bands were detected in P-5 Rov9^Sc/UnTx^ samples, whereas bands were not detected in the P-5 Rov9^Sc/DB772^ lysates, consistent with the ELISA results. Successful precipitation of protein via the phosphotungstic acid method was confirmed in Rov9^Sc/DB772^ and Rov9^Sc/UnTx^ samples, with most protein being proteinase K susceptible (D). The positions of molecular mass standards (in kilodaltons) are indicated to the left of the immunoblot (C) and gel (D).

To determine if DB772 inhibits PrP^Sc^ accumulation, microglia^Sc/DB772^, microglia^Sc/UnTx^, Rov9^Sc/DB772^, and Rov9^Sc/UnTx^ cells were assayed for PrP^Sc^ levels. The same time points that were used for BVDV detection were used for PrP^Sc^ detection. Treatment with 4 μM DB772 reduced PrP^Sc^ levels in cell lysates below detectable limits (Fig. 3A). Based on the relative minimum detection limit of the PrP^Sc^ ELISA, as determined by the standard curves, this decrease in PrP^Sc^ at P-4 is at least 1.8 logs for microglia^Sc/DB772^ cells and 2.2 logs for Rov9^Sc/DB772^ cells. After four clearance passages without DB772 (P-8) one microglia^Sc/DB772^ group and one Rov9^Sc/DB772^ group each contained minimal amounts of detectable PrP^Sc^ as determined by the standard curve (Fig. 3B).

The ELISA used in this study has been commercially validated for regulatory use and it has also previously been shown in sheep microglial cells and Rov9 cells that positive and negative ELISA results correspond appropriately with positive and negative proteinase K-resistant immunoblotting results [49]. We reconfirmed this conclusion specifically in this set of experiments by immunoblotting after DB772 treatment. At passage five, DB772-treated (4 μM) Rov9^Sc/DB772^ cell lysates lacked detectable proteinase K-resistant PrP, whereas the expected bands were detected in the Rov9^Sc/UnTx^ cell lysate (Fig. 3C). As a control for the negative immunoblot signal, electrophoresed samples of PTA-precipitated lysates stained with SYPRO Ruby demonstrate the successful precipitation of proteins (Fig. 3D), confirming the specific loss of PrP^Sc^ in the DB772-treated samples.

### DB772 effect on prion infectivity

**Figure 4:**
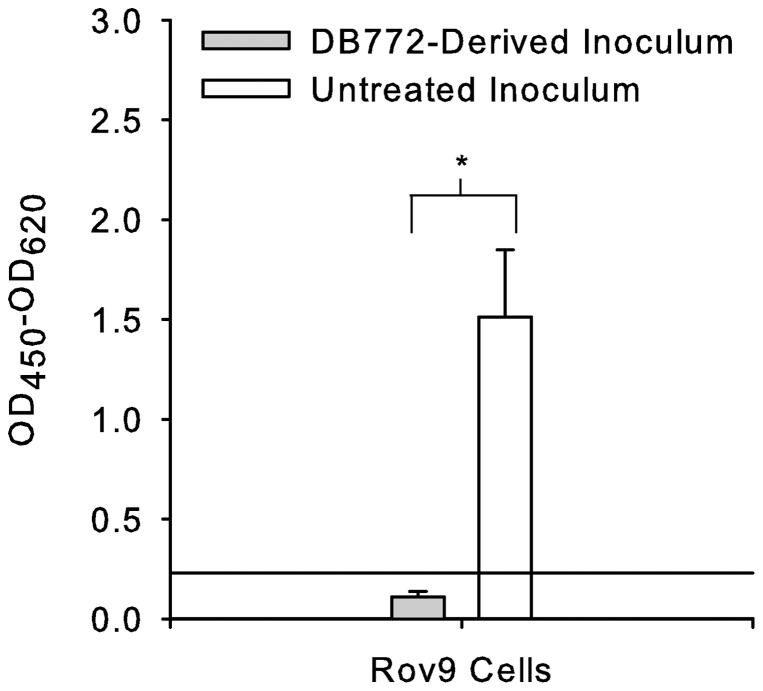
DB772 inhibits prion infectivity in Rov9 cells. Rov9^Sc/DB772^ and Rov9^Sc/UnTx^ cells were mechanically lysed and used as prion inoculum. Rov9 cells inoculated with these new inocula were then tested for PrP^Sc^ via ELISA. Data columns represent the corrected optical density (OD_450_ – OD_620_, per manufacturer's instructions to correct for nonspecific optical density) means ± one standard deviation of three independent experiments, each run in triplicate. The y-axis reference line indicates the minimum detection limit of the ELISA. *, *P*<0.001.

A prion is ultimately defined by its infectious capability; thus, to confirm that the reduced PrP^Sc^ levels correlated with reduced prion infectivity, we compared prion infectivity between Rov9^Sc/DB772^ cultures and Rov9^Sc/UnTx^ cultures (Fig. 4). Rov9 cells inoculated with the Rov9^Sc/DB772^-derived lysate failed to accumulate detectable PrP^Sc^ (0/9 were PrP^Sc^ positive); whereas, Rov9 cells inoculated with the Rov9^Sc/UnTx^-derived lysate consistently accumulated PrP^Sc^ (9/9 were PrP^Sc^ positive); thus, importantly demonstrating the loss of prion infectivity. Results are the mean of three independent treatments, each conducted in triplicate.

### DB772 effect on *PRNP* transcript and PrP^C^ expression levels

**Figure 5:**
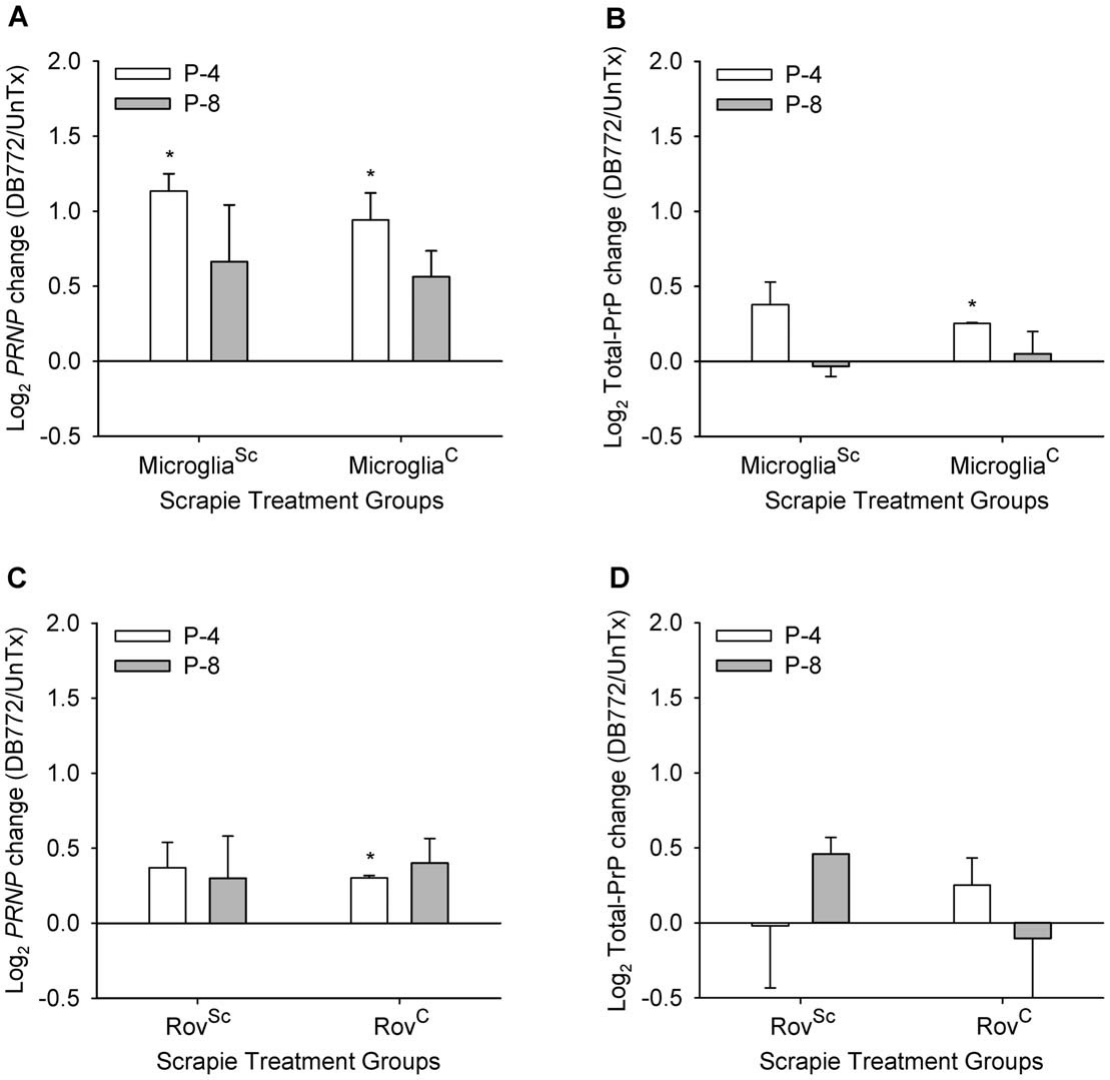
DB772 does not inhibit normal prion protein expression. Determination of DB772′s effect on *PRNP* transcript levels in primary sheep microglia (A) and Rov9 cells (C). RNA was collected at P-4 and P-8, and assayed via quantitative RT-PCR, using normalization to *GAPDH*. Columns represent the log_2_ change in DB772-treated groups compared to untreated groups from the same scrapie-treatment categories and time points (positive values indicate DB772 enhances and negative values indicate DB772 inhibits *PRNP* transcript levels). Determination of DB772′s effect on total PrP concentration in primary sheep microglia (B) and Rov9 cells (D). Total protein was collected at P-4 and P-8, and assayed for total PrP^C^ using a commercial ELISA. A standard curve was used to transform the corrected optical densities into relative concentration of PrP^C^. Columns represent the log_2_ change in DB772-treated groups compared to untreated groups from the same scrapie-treatment categories and time points. Results at each passage and for each scrapie status were statistically compared individually, using individual one-sample *t* tests, to the null hypothesis of no effect of DB772. *, *P*<0.0125.

Since PrP^C^ expression is required for PrP^Sc^ accumulation, one potential mechanism of DB772-mediated inhibition of PrP^Sc^ accumulation could be inhibition of PrP^C^ expression. *PRNP* transcript and total prion protein (PrP) levels were assayed in DB772-treated cells to determine if they were decreased as compared to untreated cells. Levels of *PRNP* transcript (Fig. 5A) and total PrP (Fig. 5B) were not decreased in microglia^Sc/DB772^ cells or in microglia^C/DB772^ cells. In fact, at P-4 the *PRNP* transcript levels are significantly elevated in microglia^Sc^ (*P* = 0.003) and microglia^C^ (*P*<0.012), although increased total PrP expression could only be verified in microglia^C^ (*P*<0.001). Similarly, no decrease in *PRNP* transcript levels was detected in Rov9 cells. The trend towards an increase in *PRNP* transcript was also evident in Rov9 cells; however, the magnitude of change was less as compared to microglial cells, and only one group (Rov^C/DB772^) showed statistical significance (Fig. 5C). Total PrP levels similarly failed to show a decrease upon DB772 treatment (Fig. 5D). In summary, expression of PrP^C^ was not inhibited in DB772-treated microglial cells or Rov9 cells.

### Concentration response of DB772 on pestivirus and PrP^Sc^

To confirm the concentration dependence of the anti-PrP^Sc^ and anti-pestiviral effects, and to compare these concentration-dependent effects, microglia^Sc^ and Rov9^Sc^ cells were exposed to a range of DB772 concentrations, and the relative levels of PrP^Sc^ and pestivirus were determined. The anti-PrP^Sc^ and anti-pestiviral effects were concentration dependent. In initial experiments (Table 2) the dynamic range for anti-pestiviral activity was determined to be 0.004–0.04 μM in microglia^Sc^ and 0.0004–0.004 μM in Rov^Sc^, whereas the dynamic range for PrP^Sc^ inhibition in microglia^Sc^ and in Rov^Sc^ was 0.4–4.0 μM (Table 2). Since the goal of this study was not to re-describe the anti-pestiviral effects of this compound [56], subsequent independent experiments focused solely on the anti-PrP^Sc^ concentration range. The final anti-PrP^Sc^ 50% tissue culture effective concentrations (TCEC_50_) for microglia^Sc^ and for Rov9^Sc^ cells were 2.4±0.2 μM and 1.9±0.4 μM, respectively (Table 2). This would represent a greater than two-log difference between the anti-PrP^Sc^ and anti-pestiviral TCEC_50_ values. In further independent experiments at higher concentrations, to describe the anti-PrP^Sc^ concentration dependence, complete anti-pestivirus activity was consistently measured at 0.4 μM (the lowest concentration used); thus, the anti-pestivirus TCEC_50_ must be lower than 0.4 μM and is different from the anti-PrP^Sc^ TCEC_50_ of 2.4 μM and 1.9 μM (microglial^Sc^ and Rov9^Sc^, respectively) (Table 2).

**Table 2:** Anti-PrP^Sc^ activity and cytotoxicity of DB772 in sheep microglial cells and Rov cells.

Cells	BVDV TCEC_50_ (μM)^a^	PrP^Sc^ TCEC_50_ (μM)*^b^*	CC_50_ (μM)*^c^*
Sheep Microglia (DB772)	.004–.04	2.4±0.2	10.6±2.3
Rov9 (DB772)	0.0004–0.004	1.9±0.4	10.5±1.4
Rov9 (curcumin)	ND*^d^*	NA*^e^*	68.6±20

a 50% tissue culture effective concentration (TCEC_50_). Values are the range in one independent experiment; two additional independent experiments confirmed the value was less than .4 μM. *^b^*Values are the mean ± one standard deviation of three independent experiments. *^c^*50% cytotoxic concentration (CC_50_). Values are the mean ± one standard deviation of four independent experiments. *^d^*Not determined. *^e^*Not applicable as no anti-PrP^Sc^ activity was measured.

### Cytotoxicity evaluation

To determine the cytotoxicity of DB772 and the potential role of cell death as the mechanism of anti-prion action, microglial and Rov9 cells were exposed to half-log dilutions of DB772. The CC_50_ was calculated to be 10.6±2.3 μM for microglial cells and 10.5±1.4 μM for Rov9 cells (Table 2). The tissue culture selectivity index (CC_50_/EC_50_) for microglia and Rov9 cells is 4.6 and 5.5, respectively. The percent cytotoxicity was determined at relevant anti-prion concentrations: the anti-PrP^Sc^ TCEC_50_ (Microglia: 2.4 μM; Rov9: 1.9 μM), and at 4 μM (concentration used in Fig. 2). Since the planned ANOVA-based analysis failed the normality assumption, the Kruskal-Wallis one-way ANOVA based on ranks, with a Tukey method of multiple comparisons was used. No change in cell viability was detected at the anti-PrP^Sc^ TCEC_50_ (Microglial % viability_2.4μM_: 98.2±2.1%; Rov9 % viability_1.9μM_: 99.5±0.5%). At 4 μM (the concentration initially used to treat the cells) no cytotoxicity was definitively detected in microglial cells; however, early cytotoxic effects are likely at this concentration (Microglial % viability_4μM_: 94.0±11%; *P* = 0.079). Significant cell death, however, was detected in Rov9 cells at 4 μM (Rov9 % viability_4μM_: 94.4±2.5%; *P*<0.001).

**Figure 6:**
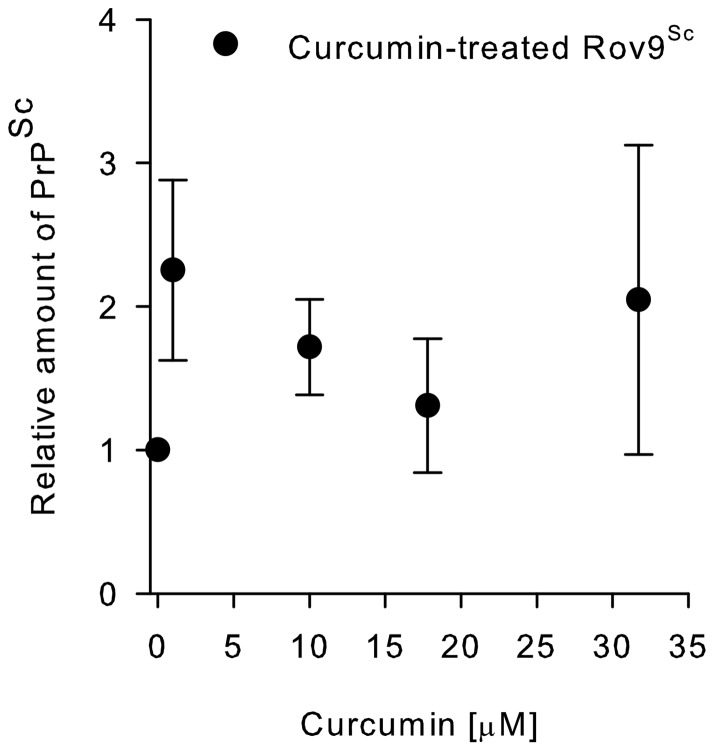
Curcumin does not inhibit PrP^Sc^ accumulation, even at cytotoxic concentrations. Rov9^Sc^ cells were exposed to a dilution series of curcumin for four days, and then an aliquot of cells was lysed for PrP^Sc^ ELISA. A standard curve was used to transform the corrected optical densities into relative concentration of PrP^Sc^. Data points represent the means ± one standard deviation of four independent experiments. Rov^Sc/UnTx^ were set to 1, and all other points were normalized to this value.

To further reduce the possibility of low levels of cytotoxicity being a non-specific cause of PrP^Sc^ inhibition, we tested the compound curcumin, which does not inhibit sheep scrapie in Rov9 cells [47]. Rov9 cells cultured in 100 and 56.4 μM of curcumin exhibited 100% cell death; thus, these samples could not be used to assay for PrP^Sc^. The calculated curcumin CC_50_ was 68.6±20 μM in Rov9 cultures. Rov9 cells cultured at 31.7 μM subjectively exhibited significant cell death and the total protein levels in the 31.7 μM-treated samples (42.7±39.7 μg/ml) were significantly (*P*<0.001) lower than all sample groups treated with less curcumin (17.8 μM: 1032 μg/ml, 10 μM: 1116 μg/ml, 1 μM: 1105 μg/ml, and 0 μM: 1097 μg/ml). No anti-PrP^Sc^ TCEC_50_ can be calculated for curcumin as there was no inhibition of PrP^Sc^ accumulation, even at the clearly cytotoxic concentration of 31.7 μM. In fact, the relative levels of PrP^Sc^ in the treated groups were actually slighter higher than the untreated groups (Fig. 6).

## Discussion

Despite previous research investigating compounds with anti-PrP^Sc^ activity [43], [44], [45], no effective chemotherapeutics exist for the treatment or prevention of prion diseases. Identification of new classes of anti-prion compounds is therefore vital, not only for the practical application of in vivo chemotherapeutics, but also for investigations studying the mechanisms of PrP^Sc^ conversion and accumulation. The data herein describe the discovery of in vitro anti-prion activity of a novel aromatic monocation. The anti-prion activity was demonstrated in two different cell culture models, including a cell type that is relevant to natural prion disease (sheep microglial cells). While the anti-prion effects described herein were discovered while using DB772 to eliminate BVDV from primary sheep microglial cells and Rov9 cells, to the authors' knowledge there are no published reports of phenyl-furan-benzimidazole cations with anti-prion activity.

The anti-PrP^Sc^ activity resulted in complete loss of PrP^Sc^ signal in sheep microglial cells and Rov9 cells by the end of the treatment (P-4), which paralleled a loss in prion infectivity. All replicates were not completely cured of PrP^Sc^, however, as after four passages without DB772 (P-8), one group from each of the microglial^Sc/DB772^ and Rov9^Sc/DB772^ samples had low levels of detectable PrP^Sc^. Regardless, these results do demonstrate significant inhibition of sheep-derived PrP^Sc^ accumulation in two cell types.

In addition to the PrP^Sc^ inhibition, DB772 treatment also inhibited BVDV in both cell lines; however, it did not cure most of the cell replicates as BVDV antigen and BVDV RNA returned to detectable levels in one microglial replicate and in all of the Rov9 cell replicates. This incomplete pestivirus inhibition is different from what was demonstrated in primary bovine fibroblasts [56]. The differing results may be due to differences in the strains of BVDV that were tested, as well as the different cell types.

The cytotoxicity of DB772 was evaluated in sheep microglial cells and Rov9 cells. The 50% cytotoxicity point was similar between sheep microglial cells and Rov9 cells (10.5 μM and 10.0 μM, respectively) and is also similar to the previously demonstrated CC_50_ of 8.6 μM in B16 melanoma cells [58]. These values are in contrast to previous cytotoxicity studies using DB772 in Madin-Darby bovine kidney (MDBK) cells, in which the CC_50_ was substantially higher at 215 μM [57]. The discrepancy between these CC_50_ values is possibly a reflection of the different cell types, but may also be a result of the different culture conditions used.

Initial investigations into the mechanism of action were conducted and while no mechanism was identified, some potential mechanisms have been ruled out. Expression of *PRNP* is required for PrP^Sc^ permissiveness [62] and the level of expression correlates with PrP^Sc^ permissiveness [63], [64]; thus, one obvious mechanism of PrP^Sc^ inhibition would be the partial to complete inhibition of *PRNP* expression. There was no evidence that DB772 inhibited *PRNP* expression, as *PRNP* transcript levels and total PrP protein levels were not decreased. In fact at passage four (the end of the DB772 treatment), microglia^Sc/DB772^ and microglia^C/DB772^ cells have significant increases in *PRNP* transcript and total PrP protein levels as compared to the untreated controls. This confirms that DB772 does not inhibit PrP^C^ expression in microglial cells and suggests that PrP^C^ expression may increase in response to DB772 exposure. While the levels of PrP^C^ were increased, it is unclear if the increase is significant enough to be biologically relevant as the magnitude of change was small (less than two fold). Similarly, there was no evidence of *PRNP* expression inhibition in Rov9 cells as the direction of changes in *PRNP* transcript levels in Rov9 cells was towards an increase in *PRNP* transcript levels with DB772 treatment, and no change was identified in total PrP levels. The difference between the sheep microglial cells and Rov9 cells regarding *PRNP* transcript levels and total PrP levels is possibly attributed to the artificial *PRNP* expression system used in Rov9 cells (i.e., tetracycline-inducible promoter), which is unlikely to respond to the same stimuli as the natural *PRNP* promoter. This highlights the importance of using a natural prion cell culture model when investigating the mechanism of action of anti-PrP^Sc^ compounds.

Another potential mechanism considered for anti-PrP^Sc^ activity was the anti-pestivirus activity. To determine if the anti-PrP^Sc^ effects were related to the anti-pestiviral effects, the concentration dependencies of anti-PrP^Sc^ and anti-pestivirus were compared in Rov9 cells. Since the purpose of this paper was not to re-describe the anti-pestiviral activity of DB772 [56], [57], the anti-pestivirus TCEC_50_ and TCEC_99_ were not fully determined for these culture models. Instead, a single experiment was used to define the range of anti-pestivirus activity and follow-up experiments confirmed maximum anti-pestivirus activity at a concentration significantly different than the anti-PrP^Sc^ TCEC_50_. The effective anti-pestivirus ranges in microglia (0.004–0.04 μM) and Rov9 (0.0004–0.004 μM) cells are similar to the anti-pestivirus EC_99_ of 0.006±0.004 μM demonstrated in cultured bovine fibroblasts [56]. Replicate experiments, which focused on the anti-PrP^Sc^ range, confirmed that the anti-pestivirus TCEC_99_ (and thus TCEC_50_) is less than 0.4 μM. In contrast, the anti-PrP^Sc^ TCEC_50_ was approximately 2 μM in either cell type. Thus, the anti-PrP^Sc^ TCEC_50_ is at least five-fold higher than the anti-pestivirus TCEC_99_. Based on previous data [56] and our initial anti-pestiviral range results, however, the difference between the anti-PrP^Sc^ TCEC_50_ and the anti-pestivirus TCEC_50_ is more likely 50–500 fold. The data demonstrate that the anti-prion and anti-pestivirus effects clearly occurred at different concentration ranges; thus, it is unlikely that the inhibition of PrP^Sc^ accumulation is mediated by a loss of BVDV infection.

Nonspecific anti-PrP^Sc^ activity associated with cell death was the final mechanism evaluated in this system. To test the effects of cytotoxicity on PrP^Sc^ accumulation, the selectivity index for DB772 was determined. Additionally, the cytotoxic and anti-PrP^Sc^ effects of curcumin, which does not inhibit PrP^Sc^ in Rov9 cells [47], were compared to DB772. DB772′s tissue culture selectivity index (SI) was 4.6 in microglia^Sc^ and 5.5 in Rov9^Sc^ cells. While the SI is low, it is similar to the SIs of many different anti-prion compounds including the often-studied quinacrine (SI: 4.5), amphotericin B (1.1), tannic acid (9.4), and cholesterol esterification modulators (1.5–11.4+). It is, however, lower than others: dextran sulfate 500 (>250) and Congo red (>17) [65]. Furthermore, the SI is similar to other less commonly studied compound classes with potentially significant anti-prion activity such as diarylthiazoles (SI: 3.3) [66]. Our curcumin data indicate that cell death does not necessarily dictate a decrease in PrP^Sc^ levels and that nonspecific effects associated with cell death do not cause the anti-PrP^Sc^ effects in this study, i.e., while the anti-prion mechanism of DB772 may or may not be closely related to its toxicity, there is no evidence that cell death is the mechanism of the inhibition. Future studies into analogues of DB772, of which there are many already available [56], [57], may identify related compounds with larger anti-PrP^Sc^ selectivity indices.

While the anti-PrP^Sc^ mechanism of action is not determined in this study, we have excluded several possibilities. One potential way to investigate the anti-PrP^Sc^ activity is to extrapolate from the anti-infectious agent mechanisms of related compounds. As an example, furamidine ([2,5-bis(4-amidinophenyl)furan]), a molecule related to DB772, demonstrates inhibition against protozoal parasites including *Plasmodium* sp. and *Trypanosoma* sp. [58]. The anti-protozoal [58] activity of the analogues of DB772, including furamidine, is thought to be mediated by DNA binding. What role nuclei acid binding may have in the DB772-mediated inhibition of PrP^Sc^ accumulation is unclear; however, it could be postulated that the DNA-binding capability results in altered transcription of genes [67], which then impacts PrP^Sc^ accumulation. Regarding DB772, its nuclear uptake is impaired relative to many other furamidine analogues [58] but little else is known about its activity. It is thus difficult at this time to speculate about any intracellular mechanism of anti-PrP^Sc^ activity of DB772.

Fortunately, due to the anti-protozoal potential of these compounds, a library of related compounds already exists. Additionally, due to the anti-pestivirus activity, in vivo work on the anti-pestiviral efficacy and pharmacokinetics has been initiated in cattle [68]. Structure-activity relationship studies are ongoing with the aims of identifying more selective anti-prion molecules, elucidating the mechanisms of action, and determining if the anti-prion activity is therapeutically or mechanistically relevant.
