# Correction: Discovery of a Novel, Monocationic, Small-Molecule Inhibitor of Scrapie Prion Accumulation in Cultured Sheep Microglia and Rov Cells

**DOI:** 10.1371/journal.pone.0119084

**Published:** 2015-03-30

**Authors:** The PLOS ONE Staff

In Table 1, the third row Reverse Primer Sequence is the same as the Forward Primer Sequence for the Target PrPC. The correct Reverse Primer Sequence should be CGCTCCATTATCTTGATGTCAGT. Please see the corrected Table 1 here.

**Table 1:** RT-PCR primer information.

Target	Forward primer sequence	Reverse Primer Sequence	Amplicon size (bp)	Reference
BVDV^a^	ATGCCCT/ATAGTAGGACTAGCA	TCAACTCCATGTGCCATGTAC	288	[60]
GAPDH^a^	GAGATGATGACCCTTTTGGC	GTGAAGGTCGGAGTCAACG	353	[61]
PrPC^b^	CCGTTACCCCAACCAAGTGT	CGCTCCATTATCTTGATGTCAGT	159	NA
GAPDH-Ov^b^	GGCGTGAACCACGAGAAGTATAA	CCCTCCACGATGCCAAAGT	120	[69]
GAPDH-Rab^b^	GCCATCACTGCCACCCAG	GAGTTTCCCGTTCAGCT	147	NA

^a^ RT-PCR primer set.

^b^qRT-PCR primer set
